# The function of our microbiota: who is out there and what do they do?

**DOI:** 10.3389/fcimb.2012.00104

**Published:** 2012-08-09

**Authors:** Noora Ottman, Hauke Smidt, Willem M. de Vos, Clara Belzer

**Affiliations:** ^1^Laboratory of Microbiology, Wageningen UniversityWageningen, Netherlands; ^2^Department of Basic Veterinary Medicine and Department of Bacteriology and Immunology, University of HelsinkiHelsinki, Finland

**Keywords:** human intestinal microbiota, functional metagenomics, metatranscriptomics, metaproteomics

## Abstract

Current meta-omics developments provide a portal into the functional potential and activity of the intestinal microbiota. The comparative and functional meta-omics approaches have made it possible to get a molecular snap shot of microbial function at a certain time and place. To this end, metagenomics is a DNA-based approach, metatranscriptomics studies the total transcribed RNA, metaproteomics focuses on protein levels and metabolomics describes metabolic profiles. Notably, the metagenomic toolbox is rapidly expanding and has been instrumental in the generation of draft genome sequences of over 1000 human associated microorganisms as well as an astonishing 3.3 million unique microbial genes derived from the intestinal tract of over 100 European adults. Remarkably, it appeared that there are at least 3 clusters of co-occurring microbial species, termed enterotypes, that characterize the intestinal microbiota throughout various continents. The human intestinal microbial metagenome further revealed unique functions carried out in the intestinal environment and provided the basis for newly discovered mechanisms for signaling, vitamin production and glycan, amino-acid and xenobiotic metabolism. The activity and composition of the microbiota is affected by genetic background, age, diet, and health status of the host. In its turn the microbiota composition and activity influence host metabolism and disease development. Exemplified by the differences in microbiota composition and activity between breast- as compared to formula-fed babies, healthy and malnourished infants, elderly and centenarians as compared to youngsters, humans that are either lean or obese and healthy or suffering of inflammatory bowel diseases (IBD). In this review we will focus on our current understanding of the functionality of the human intestinal microbiota based on all available metagenome, metatranscriptome, and metaproteome results

## Introduction

**Figure 1:**
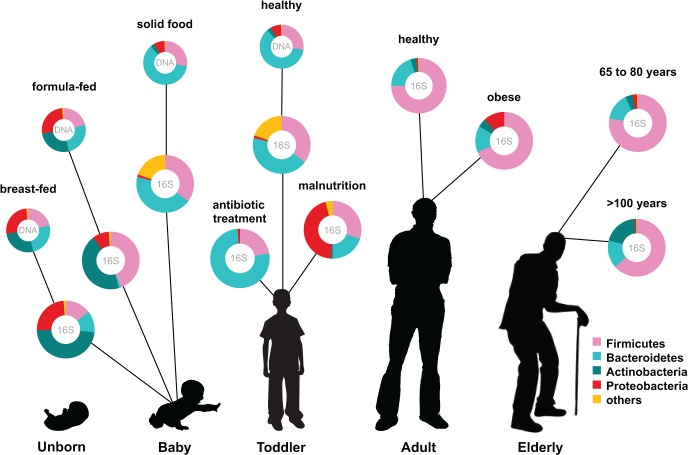
**Human microbiota: onset and shaping through life stages and perturbations.** The graph provides a global overview of the relative abundance of key phyla of the human microbiota composition in different stages of life. Measured by either 16S RNA or metagenomic approaches (DNA). Data arriving from: Babies breast- and formula-fed (Schwartz et al., 2012), baby solid food (Koenig et al., 2011), toddler antibiotic treatment (Koenig et al., 2011), toddler healthy or malnourished (Monira et al., 2011), adult, elderly, and centenarian healthy (Biagi et al., 2010), and adult obese (Zhang et al., 2009).

The human intestinal microbiota is known to play a key role in several metabolic, nutritional, physiological, and immunological processes, and recent years have seen a rapid development in the techniques for studying this previously overlooked organ (O'Hara and Shanahan, 2006). The human microbiota is established after birth and starts out as a dynamic ecosystem, dominated by bifidobacteria, that stabilizes during the first 2–3 years (Koenig et al., 2011; Scholtens et al., 2012). During life the microbial composition increases in both diversity and richness (Scholtens et al., 2012) (Figure 1) and reaches highest complexity in the human adult, with several hundred species-level phylotypes dominated by the phyla *Bacteroidetes* and *Firmicutes* (Rajilic-Stojanovic et al., 2009). Each human individual reaches a homeostatic climax composition, which likely remains relatively stable during most of a healthy adult's life. Although the individual microbial composition has an “individual core” that varies at the bacterial phylotype level and depends on the depth of the analysis (Zoetendal et al., 2008; Jalanka-Tuovinen et al., 2011), the overall phylogenetic profile can be categorized into a limited number of well-balanced host-microbial symbiotic states, the so-called enterotypes (Arumugam et al., 2011). At the late stages of life the microbiota composition becomes again less diverse and more dynamic, characterized by a higher *Bacteroides* to *Firmicutes* ratio, increase in *Proteobacteria* and decrease in *Bifidobacterium* (Biagi et al., 2010) (Figure 1).

The establishment of the bacterial ecosystem in early life is suggested to play a role in the microbial composition and disease susceptibility throughout life (Scholtens et al., 2012). A different microbiota composition is associated with chronic intestinal disorders and the severity of perturbation during disease and after antibiotic use (Sekirov et al., 2010). Diet is another important factor in microbiota composition development. Early in life there is already an impact of the diet on the microbiome: the microbiota of breast-fed and formula-fed infants was found to differ significantly in both composition and diversity. Breast-fed babies contain a microbiota that is more heterogeneous than that of formula-fed babies and contain a higher taxonomic diversity (Schwartz et al., 2012) (Figure 1). In addition, food habits can influence microbiota composition, and malnutrition results in lower abundance of *Bacteroidetes* that are shown to be specialized in breaking down the carbohydrates in energy rich western diet foods. Diet-related diseases such as allergies and obesity are also characterized by microbiota changes. Obesity is characterized by a typical *Firmicutes* to *Bacteroides* ratio. Energy harvest potential and short chain fatty acids (SCFA) are determined by the microbiota composition and have a direct effect on the host epithelial cell energy availability. A microbiota stimulated with probiotic microbes can even decrease the incidence of infant diarrhea and atopic eczema due to host immune stimulation (Niers et al., 2009; Sjogren et al., 2009).

Numerous meta-omics approaches have vastly increased the knowledge available on the genome, activity and functionality of the complex ecosystem residing in the human gut. By far the most commonly applied technique is metagenomics, which is based on direct isolation and, in most cases, sequencing of the complete genetic material obtained from an environmental sample, such as the intestine. However, one of the biggest drawbacks of this technique is its inability to display the actual metabolic activity due to the fact that it detects both expressed and non-expressed genes. In addition, it may generate information from dead cells as it is known that more than half of the cells in fecal samples are non-viable or heavily damaged (Ben-Amor et al., 2005). Instead of focusing on microbiota composition the purpose of this review is to combine the available knowledge on microbial genomics with reports on the functional metagenomics, i.e., transcriptomics and proteomics approaches. This combination is expected to provide a refined understanding of the role of the microbiota and its capabilities in regulating human health.

## Role of the microbiota in early and late life

### Early life

During natural birth, a newborn is exposed to the environmental, mainly maternal, microbiota which commences the acquisition of what we assume is a normal microbiota. The mode of delivery strongly affects the composition of the microbiota. In the case of caesarean delivery (C-section), other environmental bacteria form the basis for the microbiota instead of vaginal and faecal bacteria from the mother, reportedly resulting in a substantial reduction of bifidobacteria (Biasucci et al., 2008). In a comparison of the microbiota of babies delivered either vaginally or via C-section, it was shown that the newborns harbored undifferentiated bacterial communities across skin, oral, nasopharyngeal, and gut habitats regardless of delivery mode, and that the microbiota of C-section babies was similar to the skin communities of the mothers whereas vaginally delivered infants acquired bacterial communities resembling the vaginal microbiota of their mothers (Dominguez-Bello et al., 2010). Other factors influencing the microbiota are the type of infant feeding, gestational age, infant hospitalization, and antibiotic use by the infant. The microbiota of breast-fed infants is dominated by bifidobacteria whereas the counts of *Escherichia coli, Clostridium difficile, Bacteroides fragilis* and lactobacilli are higher in exclusively formula-fed infants (Penders et al., 2006).

The composition of the intestinal microbiota plays an important role in immune system development, and it is possible that childhood allergies are related to differences in the microbiota (Sjogren et al., 2009). The intestinal defense of the preterm infant is rather immature and exaggerates inflammatory responses that can be evoked by both commensal and pathogenic bacteria (Nanthakumar et al., 2000). Thus, the first microbes colonizing the intestinal tract hold a pivotal role. Once the core microbiota has developed, it stabilizes and is expected to become less sensitive to modification. The question is at what age does the microbiota become adult-like and recent data with large cohorts of babies in various parts of the world indicate that this is at ages after at least 3 years (Yatsunenko et al., 2012).

The succession of the microbial ecosystem in the intestinal tract of newborns is a complicated process, which is not yet fully understood. The increasing diversity of the microbiota is believed to have an effect on the functional gene content over time. Several studies have provided insight in the infant gut community structure and its perturbations during early development and highlighted the impact of weaning (Favier et al., 2002, 2003). Moreover, a recent 2.5-year case study was reported, where sixty fecal samples were collected from a healthy infant (Koenig et al., 2011) (Figure 1). The results of this study showed a gradual increase in the phylogenetic diversity of the microbiome over time. Life events such as changes in diet, illnesses, and antibiotic treatments were associated with large shifts in the abundances of major groups in this single infant. Assignment of gene functions to the metagenomic data from this study revealed an enrichment of carbohydrate-metabolizing genes involved in lactate utilization from the very beginning of life. Interestingly, during an exclusive breast-milk diet, genes facilitating the breakdown of plant-derived polysaccharides were already present, suggesting that the microbiota is metabolically prepared to receive simple plant-derived foods. This is consistent with other observations that showed high similarity in the proportions of Clusters of Orthologous Groups (COG) encoding proteins specialized for the transport and metabolism of plant polysaccharides or COGs encoding proteins transporting and metabolizing human milk oligosaccharides (HMO) between infant and maternal microbiota samples (Vaishampayan et al., 2010) (Table 1). Baby gut microbiomes are also enriched in functions involved in using glycans represented in breast milk and the intestinal mucosa, even more so in the microbiomes of Amerindian and Malawian babies compared with US babies, possibly reflecting differences in the glycan content of breast milk (Yatsunenko et al., 2012).

**Table 1:** **Percentages of COG categories expressed in the gut microbiota**.

**Population**	**Sample size (n)**	**COG categories (percentage of all genes)**							**Reference**
		**C**	**E**	**G**	**L**	**M**	**J**	**O**	
mother 1m	1	5.0	6.0	11.0	10.0	8.5	3.0	3.0	Vaishampayan et al., 2010
mother 11m	1	6.0	10.0	12.0	5.5	6.0	5.0	2.5	”
infant 1m	1	7.0	7.5	11.5	6.0	6.0	4.5	4.0	”
infant 11m	1	4.0	11.0	12.0	7.5	6.0	5.0	3.0	”
female twin pair^*a*^	2	14.0	n/a	16.0	n/a	n/a	19.0	12.0	Verberkmoes et al., 2009
healthy volunteers	10	6.0–13.0	3.5–7.0	9.5–22.0	3.5–11.0	1.5–8.0	9.0–15.0	2.5–14.0	Gosalbes et al., 2011
female cotwin (TS28)^*b*^	1	8.0	7.0	8.0	6.0	6.0	9.0	6.0	Turnbaugh et al., 2010
female cotwin (TS29)^*b*^	1	8.0	6.0	9.0	5.0	5.0	10.0	7.0	”
female cotwin (TS28)^*c*^	1	5.0	6.0	8.0	9.0	6.0	7.0	5.0	”
female cotwin (TS29)^*c*^	1	5.0	7.0	9.0	9.0	6.0	6.0	4.0	”

COG descriptions: C, Energy production and conversion; E, Amino acid transport and metabolism; G, Carbohydrate transport and metabolism; L, DNA replication, recombination, and repair; M, Cell envelope biogenesis, outer membrane; J, Translation, ribosomal structure, and biogenesis; O, Post-translational modification, protein turnover, chaperones

a out of the core proteome

b out of genes with high relative expression

c out of genes with low relative expression.

Recently it has been shown that the use of specific human milk-derived glycans such as HMO utilization is not exclusive to certain well-known infant colonizers, such as *Bifidobacterium* species, since members of the genus *Bacteroides* can also use milk glycans as a sole carbon and energy source (Marcobal et al., 2010). Moreover, it has been shown that *Bacteroides thetaiotaomicron* responds to common structural motifs found in oligosaccharides from mother's milk and intestinal mucin glycans, suggesting that HMOs may mimic mucus glycans to attract mucin-adapted resident mutualists to an infant microbiota (Marcobal et al., 2011). However, specific HMO components select for HMO-adapted species such as *Bifidobacterium longum* subsp. *infantis*, and provide a selective advantage to this species *in vivo* when biassociated with *B. thetaiotaomicron* in the gnotobiotic mouse gut. The complex oligosaccharide mixture within HMOs thus attracts both mutualistic mucus-adapted species and HMO-adapted bifidobacteria to the infant intestine that likely facilitate both milk and future solid food digestion.

Little is known of the effect of diet on the composition and in particular the activity of the developing gut microbiota. Comparison of host epithelial cell gene expression and microbiota profile between breast- and formula-fed infants demonstrated that differences in the diet of infants can have an influence on the host gene expression via the gut microbiota (Schwartz et al., 2012). Virulence characteristics of the microbiota were the only functional properties that were found to differ among these two groups. Further analysis of the host transcriptome revealed a subset of eleven immunity and mucosal defense-related genes exhibiting evidence of a multivariate relationship with microbiome virulence characteristics. This provides additional proof for the capability of human milk to promote the mutualistic interactions between the mucosal immune system and the microbiome in maintaining intestinal homeostasis. Gene content analysis of the gut microbiome of 110 individuals including both adults and babies from Venezuela, Malawi, and the US revealed age-related changes in the metabolism of vitamins B12 (cobalamin) and folate (Yatsunenko et al., 2012). Genes involved in *de novo* biosynthesis of folate decreased with age whereas genes encoding most enzymes associated with cobalamin biosynthesis increased, correlating with previous data of blood levels of these vitamins in different age groups (Monsen et al., 2003).

The key players in the neonate gut are the bifidobacteria, which dominate the microbial community of human milk-fed infants. A number of studies using metagenomic approaches have also demonstrated the importance of this genus in the developing gut (Turroni et al., 2012; Yatsunenko et al., 2012), while at the same time other studies have reported low abundance or even absence of bifidobacteria (Palmer et al., 2007; Koenig et al., 2011), most likely due to technical biases related to DNA extraction protocols or the selected PCR primers. Genome analysis of *Bifidobacterium longum* subsp. *infantis* revealed a nutrient-utilization strategy targeting milk-derived molecules which are not of nutritional value to the infant (Sela et al., 2008). The proteomic profile of the organism grown on HMOs confirmed the activity of these genes (Sela et al., 2008). This suggests *B. longum* subsp. *infantis* coevolved with its infant host and under the presence of human milk compounds.

Furthermore, the type of milk, either mother's milk or formula, determines the colonization with different types of bifidobacteria. Breast-fed infants contain a high abundance of *Bifidobacterium breve*. In contrast, faecal samples from standard formula-fed infants lacked detectable amounts of this *B. breve* but contained *B. longum*. Remarkably, infants that received breast milk and later a prebiotic formula consisting of a standard formula milk containing a mixture of specific galacto- and fructo-oligosaccharides, continued to harbor a *B. breve*-dominant faecal population (Boesten et al., 2011).

Transcriptional analysis of the response of *B. longum* to human milk and formula milk indicated upregulation of genes involved in carbohydrate metabolism in breast milk, endorsing the concept that the bifidogenic effect of breast milk is primarily based on its oligosaccharides (Gonzalez et al., 2008). Moreover, the same study found upregulation of putative genes for cell surface type 2 glycoprotein-binding fimbriae associated with attachment and colonization in the intestine in both breast milk and formula milk when compared to semisynthetic medium with glucose. Transcriptome analysis of *B. breve* in a mouse model also showed differential expression of genes encoding for the production of type IVb tight adherence pili, which are essential for efficient *in vivo* murine gut colonization (O'Connell Motherway et al., 2011).

Another study comparing the bifidobacterial transcriptome of breast-fed infants and prebiotic-containing formula-fed infants showed that in the beginning of the intervention breast-fed infants had higher counts of bifidobacteria compared to the formula-fed infants (Klaassens et al., 2009). However, during the intervention the bacterial numbers and species diversity of *Bifidobacterium* increased significantly in the formula-fed infants, possibly on account of the galacto- and fructo-oligosaccharides in the formula. These prebiotics have also previously been shown to shift the bifidobacterial quantities toward those of breast-fed infants (Knol et al., 2005). The metatranscriptome analysis in babies revealed that the most prominent functions of the transcripts were related to carbohydrate metabolism, with higher expression of genes encoding these functions in breast-fed infants compared to formula-fed infants (Klaassens et al., 2009). This included significant expression of genes involved in HMO degradation. Moreover, the expression of genes involved in folate production was observed in all babies indicating that intestinal bifidobacteria produced this important vitamin involved in neural development. In the same study, a gene for bifidobacterial transaldolase, which is a key enzyme of the non-oxidative phase of the pentose phosphate pathway, was expressed in samples from all infants. Bifidobacterial transaldolase was also found in the only metaproteome study thus far to look at the infant gut microbiota (Klaassens et al., 2007). Production of the protein spot on a 2D-gel corresponding to this protein was increased over time, suggesting an increase in the numbers and activity of bifidobacteria in the infant's gut. Understanding the factors relating to the existence and host interactions of bifidobacteria and linking the functionality of this early intestinal colonizer to specific diets and groups of healthy or diseased individuals may eventually lead to the possibility of guiding the development of the microbiota. This can be achieved with pro- and prebiotic supplemented infant formulas that are aimed at increasing the bacterial diversity and a more optimal bifidobacterial community composition.

### Late life

In addition to the beginning of life, the microbiota also undergoes significant changes toward the other extremity of life, old age. These alterations, however, are not clear-cut partially due to the various physiological changes that the elderly go through. These include factors such as modifications in lifestyle, nutritional behavior, increase in infection rates and inflammatory diseases, and therefore the need for more medication. All of these issues will certainly also affect the composition and activity of the microbiota, but the course and mechanisms behind these changes are not yet completely understood.

The process of ageing has been demonstrated to have a negative effect on the diversity of the microbiota, but different studies have reported conflicting results on the age-related changes with regard to the two major phylogenetic groups. Assessment of the gut microbiota of the elderly with quantitative PCR revealed high levels of *Escherichia coli* and *Bacteroidetes* as well as a significant difference in the *Firmicutes* to *Bacteroidetes* ratio for adults (10.9) and elderly individuals (0.6) (Mariat et al., 2009). In this study the total bacterial counts for adults and seniors were comparable whereas another study, employing cytosine (%G + C) profiling and 16S rRNA gene sequencing, described a significant reduction in overall numbers of microbes in elderly subjects compared to young adults (Makivuokko et al., 2010). They also observed lower numbers of *Firmicutes* and an increase in *Bacteroidetes*, with lowered amounts of known butyrate producers belonging to *Clostridium* cluster XIVa.

Another study, which included young (20–40 years old), elderly (60–80 years old) and an additional group of centenarian citizens (~100 years old), clearly demonstrated that the process of ageing coincides with decreasing microbiota diversity (Biagi et al., 2010) (Figure 1). By using the Human Intestinal Tract Chip (HITChip) and qPCR, they observed that the composition of microbiota was quite similar between the young and the elderly groups represented by dominant portions of *Firmicutes* and *Bacteroidetes* (95% of total bacteria). The centenarian group also showed a dominant portion of *Firmicutes* and *Bacteroidetes* (93% of total bacteria). The *Firmicutes/Bacteroidetes* ratios obtained for the centenarians, elderly and young adults were 3.6, 5.1, and 3.9, respectively. However, there was a significant decrease in the *Firmicutes* subgroup *Clostridium* cluster XIV and an increase in *Bacilli* in the centenarian group. Furthermore, there was a significant increase in several facultative anaerobes, members of the *Proteobacteria* phylum, many of which constitute opportunistic pathogens. This rearrangement of the microbiota does not seem to be in favor of the aging subjects that showed an increased level of circulating inflammatory cytokines. These were inversely associated with bacteria belonging to *Clostridium* cluster XIV and *Clostridium* cluster IV that include the main butyrate-producers in the gut. Butyrate has been associated with a range of health effects from anti-inflammatory properties to enhancement of intestinal barrier function (Macfarlane and Macfarlane, 2011).

Recently, pyrosequencing of tagged PCR-amplified 16S rRNA genes was applied to characterize the fecal microbiota of 161 seniors aged 65 years and older in the ELDERMET consortium (Claesson et al., 2011). In this extensive study the elderly microbiota was observed to be dominated by the phylum *Bacteroidetes* (57%) compared with *Firmicutes* (40%). However, the proportions of the major phyla showed extraordinary variation between individuals, with the proportion of *Bacteroidetes* ranging from 3 to 92% and *Firmicutes* from 7 to 94%. In addition to the general composition, also the core microbiota of the elderly differed substantially with that of young adults, characterized by a shift to a more *Clostridium* cluster IV-dominated community in the elderly. The microbiota of the elderly showed temporal stability for the majority of subjects as revealed by analysis of 3-month follow-up samples.

These studies indicate that there undoubtedly are fluctuations in the elderly microbiota, but both the threshold for an “aged” microbiota and the trends for these changes seem to be highly variable. Some of these differences may be explained by country-specific dietary habits, as the most recent studies used separate cohorts from two different European countries, Italy (Biagi et al., 2010) and Ireland (Claesson et al., 2011). The living environments of elderly people are highly dependent on their health status, with healthier seniors living independently and subjects with medical issues often living in nursing homes. These factors can also influence the aging gut microbiota. Follow-up studies assessing the function of the elderly gut microbiota by functional metagenomic techniques already applied for the infant and adult microbiota will shed more light on these issues and reveal prospects for possible dietary interventions aimed at improving the health of the elderly.

## Microbiota activity in response to diet

Host dietary habits appear to affect gut microbiota composition, but the actual association between different diets and the microbial community composition as well as the underlying causes for this are still unclear. Although there was no clear environmental or genetic explanation found for the initial clustering of the enterotypes (Arumugam et al., 2011), these were found to be strongly associated with long-term diets, with protein and animal fat correlating with the enterotype characterized by high levels of *Bacteroidetes*, and carbohydrates with the *Prevotella* enterotype (Wu et al., 2011). Differences in microbiota composition as a result of diverging dietary habits was also shown in a comparison of the microbiota of European children, who consumed a diet high in animal protein, sugar, starch and fat and low in fiber, and children from Burkina Faso, where the predominantly vegetarian diet consists mainly of carbohydrates, fiber and non-animal protein (De Filippo et al., 2010). The European microbiome was enriched with *Firmicutes* and *Proteobacteria*, whereas *Actinobacteria* and *Bacteroidetes* were more represented in the African children. Interestingly, *Xylanibacter* and *Prevotella* were only present in the children from Burkina Faso, leading the authors to hypothesize that members of these genera could improve the ability to extract calories from indigestible polysaccharides commonly consumed in rural Africa indicating a coevolution of the microbial community with the polysaccharide-rich diet. Malnourished children from poor socio-economic status families in Bangladesh were found to have lower diversity of gut microbiota compared to healthy children from moderate to high income families in the same region, characterized by lower relative abundance of *Bacteroidetes* and a dominance of *Proteobacteria* (Monira et al., 2011). The authors suggest that the low presence of *Bacteroidetes*, which are known to digest complex dietary material and thus improve energy extraction from various foods, and the higher presence of potentially pathogenic *Proteobacteria* might contribute to explaining the poor health of the malnourished children.

In a metagenome study, short-term dietary intervention (high-fat/low-fiber or low-fat/high-fiber diets) lead to rapid changes in the microbiome composition but was not sufficient to shift individuals between the two enterotypes described in the same study (Wu et al., 2011). Few functional gene categories, including bacterial secretion system, protein export, and lipoic acid metabolism, differentiated between the two test diets suggesting a shift in selected bacterial functions in response to the dietary changes. Microbiome analysis of subjects on a diet rich in protein, typically consumed in the US, showed enrichment of multiple Enzyme Commission (EC) groups when compared with Malawian and Amerindian subjects consuming a diet high in carbohydrates (Yatsunenko et al., 2012). These included degradation of glutamine and other amino acids, catabolism of simple sugars, vitamin biosynthesis, and bile salt metabolism. Degradation of glutamine has earlier been found to be overrepresented in carnivorous mammalian microbiomes, while glutamate synthase, which was enriched in Malawian/Amerindian microbiomes, was present in higher proportions in herbivorous mammalian microbiomes (Muegge et al., 2011).

Several metatranscriptome and metaproteome studies describing the human intestinal microbiota have confirmed the importance of bacterial functions related to carbohydrate metabolism in the colon. Enrichment of these genes has earlier been shown in metagenomic studies of the human gut (Gill et al., 2006; Kurokawa et al., 2007; Turnbaugh et al., 2009a). Metatranscriptome analysis of fecal samples from two healthy volunteers found that most expressed genes (26% of all sequenced and annotated transcripts) were involved in the metabolism of carbohydrate (Booijink et al., 2010). Recently the majority of bifidobacterial transcripts within the fecal community of adults were also reported to be involved in metabolism of carbohydrates of plant origin (Klaassens et al., 2011).

Similar results were seen in a transcriptional analysis of fecal samples from a monozygotic, obese twin pair (Turnbaugh et al., 2010) (Table 1), and metatranscriptomics analysis of fecal samples from ten healthy volunteers (Gosalbes et al., 2011) (Table 1). Metatranscriptomic data from the less studied small intestinal microbiota showed enrichment in sugar phosphotransferase (PTS) and other carbohydrate transport systems, as well as energy- and central metabolic, and amino acid conversion pathways as compared with the metagenome (Zoetendal et al., 2012). This suggests rapid uptake and fermentation of available simple sugars by the small intestinal microbiota, compared to the degradation of more complex carbohydrates by the bacteria in the colon. The importance of carbohydrate metabolism is also evident from the enormous amount of carbohydrate-active enzymes (CAZymes) present in the gut microbiome. By applying a multi-step functional screening procedure of a metagenomic library from the feces of volunteer following a fiber-rich diet, 73 CAZymes from 35 different families were recently discovered (Tasse et al., 2010).

Shotgun metaproteomics approach used to identify microbial proteins in fecal samples from a female twin pair identified several COG categories more highly represented in the microbial metaproteome compared to the average metagenome (Verberkmoes et al., 2009) (Table 1). A high proportion of the proteins that were equally abundant in both samples were from common gut bacteria, such as *Bacteroides, Bifidobacterium*, and *Clostridium*. These included proteins involved in translation, carbohydrate metabolism and energy production. In another study, two human fecal samples were analyzed and the functions of the identified proteins were predicted (Rooijers et al., 2011). The most abundantly present COGs were involved in translation, energy production, and conversion as well as carbohydrate transport and metabolism, which supports the findings of studies linking the microbiota with carbohydrate metabolism (Kovatcheva-Datchary et al., 2009). The study also pointed out the abundance of *Akkermansia muciniphila*, the only intestinal member of the *Verrucomicrobia*, within the microbiota and showed that most of the proteins produced by these bacteria are involved in carbohydrate transport and metabolism as well as amino acid transport and metabolism. This is in line with observation that *A. muciniphila* can use mucin as the sole carbon and nitrogen source (Derrien et al., 2008). The fecal samples were also subject to metagenome sequencing and the phylogenetic diversity was determined with two approaches, 16S rRNA sequence analysis of the metagenomic data sets and an abundance analysis of the metagenomic sequences using a synthetic metagenome as reference set. The results showed that *Bacteroidetes, Firmicutes, Actinobacteria, Verrucomicrobia*, and *Proteobacteria* were the dominant groups in the microbiota of the study subjects.

These results were further confirmed by analysing the gut metaproteome of three healthy subjects over a period of 6–12 months (Kolmeder et al., 2012). In this study, proteins involved in carbohydrate transport and metabolism accounted for over 10% of the detected proteins, forming a part of the core metaproteome found in all the test subjects. The glycolysis pathway, in particular, was noticeable with several related enzymes identified. After assigning the spectral hits for each COG functional category per phylum, it was apparent that *Firmicutes* and *Actinobacteria* were responsible for the active carbohydrate metabolism, while *Bacteroidetes* showed more mixed functions. Both *Firmicutes* and *Bacteroidetes* were found to have an active carbohydrate metabolism on a transcriptional level in an earlier report (Gosalbes et al., 2011). Furthermore, Kolmeder et al. (2012) observed that the majority of the identified actinobacterial peptides were predicted to be involved in sugar metabolism. The importance of carbohydrate metabolism has been shown also previously for the core genome of bifidobacteria (Bottacini et al., 2010). Temporal analysis showed that the metaproteome is stable over time, as is the microbial composition of the gut, suggesting that homeostasis in function and composition of the intestinal microbiota are tightly linked (Kolmeder et al., 2012).

Recently, a metatranscriptomics approach with RNAseq has been applied to investigate the effect of a fermented milk product (FMP) containing several probiotics on the gut microbiome of gnotobiotic mice colonized with a model human gut microbiota and monozygotic twins (McNulty et al., 2011). There were no or minimal changes observed in the bacterial species composition in mice and humans after consumption of FMP. Still, transcriptional analysis revealed significant changes in numerous metabolic pathways, especially in carbohydrate metabolism, in both mice and human subjects. The question, however, is whether this reflects a functional difference in the colon or is a result of technical or biological effects such as variations in the transit time of the fecal material used for this analysis.

Metagenomic approaches combined with studies using gnotobiotic animals colonized with only a few known microorganisms or even the entire human fecal microbiota provide a powerful tool for examining the relationship between the host and the functionality of the microbial community under controlled conditions. A study of humanized gnotobiotic mice transplanted with either fresh or frozen adult human fecal microbial communities into germ-free C57BL/6J mice revealed a stable and heritable colonization which enabled a diet intervention, where the mice were switched from a low-fat, plant polysaccharide to a high-fat, high-sugar diet (Turnbaugh et al., 2009b). This diet change induced a structural shift in the microbiota within one day and presented an enrichment for various KEGG pathways involved in nutrient processing compared to the control diet. Metatranscriptome analysis of rRNA-depleted RNA isolated from the ceca of the humanized mice demonstrated a clear difference in the gene expression of the mice on the Western diet compared to the control group, with upregulation of clusters containing *Clostridium innocuum* strain SB23 genes encoding Western diet-associated transcripts (pyruvate formate-lyase, PTS, phosphoglycerate kinase) and *Firmicutes* gene clusters encoding ABC-type sugar transport systems.

A shift in the microbial community was also seen after switching both wild-type and RELMβ-deficient mice to a high-fat diet, indicating that the diet itself was responsible for the detected changes independent of obesity (Hildebrandt et al., 2009). RELMβ is a colonic goblet cell-specific gene, whose expression is dependent on the presence of the gut microbiome. After the dietary switch the amounts of *Proteobacteria, Firmicutes*, and *Actinobacteria* increased whereas *Bacteriodetes* decreased, as measured from fecal samples. Analysis of gene functions revealed a decrease in the number of metabolic genes under the high-fat condition, possibly as a result of nutrient deficiency. However, as also noted by Turnbaugh et al. (2009b), a group of genes for ABC-transporters increased in abundance, indicating adaptation to the high-fat diet by enhancing nutrient intake in an environment with limited substrate availability.

Mice colonized with 10 sequenced human gut bacteria, and fed with a series of refined diets showed that casein concentration was highly correlated with the yield of total DNA per fecal pellet in all 17 test diets (Faith et al., 2011). The abundance of all of the ten species was significantly associated with casein, with seven of them positively correlated with casein concentration and three negatively correlated. None of the diets caused significant changes in the gene expression of the bacterial species, analyzed by RNA-sequencing, but high expression of genes predicted to be involved in pathways using amino acids as substrates for nitrogen, as energy and/or carbon sources were found for the species positively correlated with casein.

In conclusion, the studies to date endorse the concept that the intestinal microbiota thrives on using polysaccharides and peptides, which are indigestible to human (Guarner and Malagelada, 2003). The metagenomic data are confirmed on a functional level by the metatranscriptomics and metaproteomics data. The composition of the microbiota in the colon is dominated by *Firmicutes* that appear to be active in carbohydrate metabolism whereas *Bacteroidetes* show activity in a number of functions like energy production and conversion as well as amino acid transport and metabolism, in addition to carbohydrate metabolism. The complex polysaccharides are degraded by a specialized microbial community and the released oligosaccharides can in turn be used by other commensal bacteria. In this manner, diet is has a crucial influence on the intestinal microbial activity.

## Microbial imbalances and disease

### Inflammatory bowel diseases

The gut microbiota has been connected to several diseases, with obesity and inflammatory bowel diseases (IBD) representing the most studied disorders to date. Most research about potential differences of microbiota related to different disease states has so far focused on describing the composition and diversity of the microbiome in patients compared to healthy subjects, and consequently revealing interesting associations between them. In order to get a better understanding of the underlying mechanisms of the relationship between the microbial communities and specific disorders, functional microbiomic approaches need to be employed.

Despite exhaustive research efforts, the etiology and pathogenesis of IBD, including Crohn's disease (CD) and ulcerative colitis (UC) have stayed unclear. The causes of these intestinal diseases are most likely linked with both human gene- and microbiome-associated factors (Pflughoeft and Versalovic, 2012). CD and UC patients seem to harbor separate microbial communities both from each other and healthy subjects, and also have lower bacterial diversity compared to healthy people (Manichanh et al., 2006; Dicksved et al., 2008; Qin et al., 2010). Several bacterial groups have been implied to be either increased or decreased in association with IBD. However, it is not clear whether this dysbiosis is the reason for the inflammation in IBD, or simply something caused by the disturbed environment in the GI tract.

Metagenomic studies and microarray analyses have demonstrated a reduction of *Firmicutes*, such as *Faecalibacterium prausnitzii*, in CD (Manichanh et al., 2006; Kang et al., 2010). A 16S rRNA gene pyrosequencing study of twin pairs who were concordant or discordant for CD or UC showed a clear division in the microbial composition between CD and healthy individuals but not between UC and healthy individuals (Willing et al., 2010). There were more *Firmicutes* detected for colonic involvement CD and less for ileum localized CD (ICD) compared to healthy subjects. In addition to *F. prausnitzii*, also other core members of the microbiota, such as *Roseburia*, were less abundant in ICD. Interestingly, a separate study analyzing the same samples showed clear shifts in metabolic profiles corresponding to the same bacterial groups (Jansson et al., 2009). Pathways with differentiating metabolites included those involved in the metabolism and or synthesis of amino acids, fatty acids, bile acids, and arachidonic acid.

A recent analysis of the fecal microbiota of UC patients in relapse and remission further confirmed the reduction of bacterial diversity in these patients and showed that this mainly affects members of the *Clostridium* cluster IV within the phylum Firmicutes (Rajilic-Stojanovic et al., 2012). The authors also speculated on the role of SCFA in UC as they reported reduced numbers of butyrate-producing bacteria, along with other studies (Frank et al., 2007), and a disturbed abundance of typical propionate producers. A depletion of one propionate producer, *A. muciniphila*, was observed in the fecal samples while another one, *Megamonas* sp. was increased. *A. muciniphila* was previously found to be decreased in biopsies of patients with UC with an associated increase in *Ruminococcus* sp. (Png et al., 2010). The role of butyrate and propionate, both of which have anti-inflammatory properties (Tedelind et al., 2007), in UC is still under debate (Chapman et al., 1994; Roediger et al., 1997). These and forthcoming studies will eventually help in screening and diagnosing IBD patients.

### Obesity and metabolic syndrome

Obesity and obesity-associated metabolic disorders, such as metabolic syndrome and type 2 diabetes have been suggested to be associated with the composition and function of the intestinal microbiota. Initial research showed an increase in the relative abundance of *Firmicutes* and decrease in *Bacteroidetes* in both obese mice (Ley et al., 2005) and humans (Ley et al., 2006), but later studies have failed to endorse these findings and showed inconsistent results with respect to the changes in the microbiota of obese people (Nadal et al., 2009; Santacruz et al., 2009, 2010; Zhang et al., 2009; Schwiertz et al., 2010) (Figure 1). In addition, the transfer of the gut microbiota of obese (*ob/ob*) mice to germ-free wild-type mice causes an increase in fat mass in the recipients, indicating that the obese microbiota has an increased capacity to harvest energy from the diet (Turnbaugh et al., 2006).

Departing from these findings, scientists are now trying to unravel the mechanisms behind the observations. One study found that loss of Toll-like receptor (TLR) 5, which is a transmembrane protein recognizing bacterial flagellin, in a mouse model results in a phenotype resembling human metabolic syndrome (Vijay-Kumar et al., 2010). The authors speculated that the loss of this receptor alters the microbiota inducing low-grade inflammatory signalling, which eventually leads to hyperphagia and metabolic syndrome. In another study, TLR 2-deficient mice, which are protected from diet-induced insulin resistance under germ-free settings, developed a condition reminiscent of metabolic syndrome after colonization (Caricilli et al., 2011). The microbiota of the mice showed notable increase in *Firmicutes* and slight increase in *Bacteroidetes* compared to controls. The authors suggested that the mechanisms by which the TLR 2-deficient mice became insulin resistant and, later, obese could be related to increased capacity for energy harvesting from the diet or alternatively to increased level of LPS caused by increased gut permeability and LPS absorption. Recently, it was shown that antibiotic treatment with vancomycin for diet-induced obese mice significantly reduced the proportions of *Firmicutes* and *Bacteroidetes*, and increased *Proteobacteria* (Murphy et al., 2012). These changes were associated with improvement in the metabolic abnormalities associated with obesity, by reducing body weight gain and improving inflammatory and metabolic health of the host. Based on these studies, it seems plausible that the ability of the gut microbiota to regulate inflammatory responses play an important role in the complex mechanisms behind obesity and metabolic syndrome. Still, more long-term studies in animal models and humans are required to acquire a clearer picture of the relationship between the intestinal microbiota and different diseases.

## Concluding remarks

The complexity of the microbiota–host interactions has been the prime obstacle in defining microbial functionality at a post-genomic level. The recent technical advances in analyzing genomes, transcriptomes and proteomes of complex bacterial consortia and intra- and interspecies metabolic networks help to tackle this problem and will enable systems-level analyses of the crosstalk between the microbiota and the host.

There are multiple reports providing circumstantial evidence to support the concept that microbiota composition and activity influence host metabolism and disease development. These examples include the differences in microbiota composition and microbiota expressed proteins of breastfeeding as compared to formula-fed babies (Schwartz et al., 2012), differences between microbiota composition and activity between healthy and malnourished infants (Monira et al., 2011), differences in the microbiota composition of elderly and centenarians as compared to youngsters (Biagi et al., 2010), and differences in microbiota composition and activity between humans that are either lean or obese (Ley et al., 2005; Zhang et al., 2009) and healthy or suffering of IBD (Willing et al., 2010). The data suggest that the activity and composition of the microbiota is affected by food intake and genetic background of the host. Most findings are supported by animal studies but there is also data on human subjects. The field of functional microbiomics is still rapidly advancing with continuously emerging new techniques and results. Nevertheless, a lot of times the high throughput techniques fail to correlate bacterial species and genome content to function due to the lack of characterized isolates and genes. It is important to identify the regulating parameters of the functioning intestinal ecosystem to gain insight into the influence of the microbiota on human development, aging, and disease.

### Conflict of interest statement

The authors declare that the research was conducted in the absence of any commercial or financial relationships that could be construed as a potential conflict of interest.

## References

[B1] Arumugam M. Raes J. Pelletier E. Le Paslier D. Yamada T. Mende D. R. Fernandes G. R. Tap J. Bruls T. Batto J. M. Bertalan M. Borruel N. Casellas F. Fernandez L. Gautier L. Hansen T. Hattori M. Hayashi T. Kleerebezem M. Kurokawa K. Leclerc M. Levenez F. Manichanh C. Nielsen H. B. Nielsen T. Pons N. Poulain J. Qin J. Sicheritz-Ponten T. Tims S. Torrents D. Ugarte E. Zoetendal E. G. Wang J. Guarner F. Pedersen O. De Vos W. M. Brunak S. Dore J. Meta H. I. T. C. Antolin M. Artiguenave F. Blottiere H. M. Almeida M. Brechot C. Cara C. Chervaux C. Cultrone A. Delorme C. Denariaz G. Dervyn R. Foerstner K. U. Friss C. Van De Guchte M. Guedon E. Haimet F. Huber W. Van Hylckama-Vlieg J. Jamet A. Juste C. Kaci G. Knol J. Lakhdari O. Layec S. Le Roux K. Maguin E. Merieux A. Melo Minardi R. M'rini C. Muller J. Oozeer R. Parkhill J. Renault P. Rescigno M. Sanchez N. Sunagawa S. Torrejon A. Turner K. Vandemeulebrouck G. Varela E. Winogradsky Y. Zeller G. Weissenbach J. Ehrlich S. D. Bork P. (2011). Enterotypes of the human gut microbiome. Nature 473, 174–180 10.1038/nature09944 21508958 PMC3728647

[B2] Ben-Amor K. Heilig H. Smidt H. Vaughan E. E. Abee T. De Vos W. M. (2005). Genetic diversity of viable, injured, and dead fecal bacteria assessed by fluorescence-activated cell sorting and 16S rRNA gene analysis. Appl. Environ. Microbiol. 71, 4679–4689 10.1128/AEM.71.8.4679-4689.2005 16085863 PMC1183343

[B3] Biagi E. Nylund L. Candela M. Ostan R. Bucci L. Pini E. Nikkila J. Monti D. Satokari R. Franceschi C. Brigidi P. De Vos W. (2010). Through ageing, and beyond: gut microbiota and inflammatory status in seniors and centenarians. PLoS ONE 5:e10667 10.1371/journal.pone.0010667 20498852 PMC2871786

[B4] Biasucci G. Benenati B. Morelli L. Bessi E. Boehm G. (2008). Cesarean delivery may affect the early biodiversity of intestinal bacteria. J. Nutr. 138, 1796S–1800S 18716189 10.1093/jn/138.9.1796S

[B5] Boesten R. Schuren F. Ben Amor K. Haarman M. Knol J. De Vos W. M. (2011). Bifidobacterium population analysis in the infant gut by direct mapping of genomic hybridization patterns: potential for monitoring temporal development and effects of dietary regimens. Microb. Biotechnol. 4, 417–427 10.1111/j.1751-7915.2010.00216.x 21375714 PMC3818999

[B6] Booijink C. C. Boekhorst J. Zoetendal E. G. Smidt H. Kleerebezem M. De Vos W. M. (2010). Metatranscriptome analysis of the human fecal microbiota reveals subject-specific expression profiles, with genes encoding proteins involved in carbohydrate metabolism being dominantly expressed. Appl. Environ. Microbiol. 76, 5533–5540 10.1128/AEM.00502-10 20562280 PMC2918960

[B7] Bottacini F. Medini D. Pavesi A. Turroni F. Foroni E. Riley D. Giubellini V. Tettelin H. Van Sinderen D. Ventura M. (2010). Comparative genomics of the genus Bifidobacterium. Microbiology 156, 3243–3254 10.1099/mic.0.039545-0 20634238

[B8] Caricilli A. M. Picardi P. K. De Abreu L. L. Ueno M. Prada P. O. Ropelle E. R. Hirabara S. M. Castoldi A. Vieira P. Camara N. O. Curi R. Carvalheira J. B. Saad M. J. (2011). Gut microbiota is a key modulator of insulin resistance in TLR 2 knockout mice. PLoS Biol. 9:e1001212 10.1371/journal.pbio.1001212 22162948 PMC3232200

[B9] Chapman M. A. Grahn M. F. Boyle M. A. Hutton M. Rogers J. Williams N. S. (1994). Butyrate oxidation is impaired in the colonic mucosa of sufferers of quiescent ulcerative colitis. Gut 35, 73–76 8307454 10.1136/gut.35.1.73 PMC1374636

[B10] Claesson M. J. Cusack S. O'Sullivan O. Greene-Diniz R. De Weerd H. Flannery E. Marchesi J. R. Falush D. Dinan T. Fitzgerald G. Stanton C. Van Sinderen D. O'Connor M. Harnedy N. O'Connor K. Henry C. O'Mahony D. Fitzgerald A. P. Shanahan F. Twomey C. Hill C. Ross R. P. O'Toole P. W. (2011). Composition, variability, and temporal stability of the intestinal microbiota of the elderly. Proc. Natl. Acad. Sci. U.S.A. 108(Suppl. 1), 4586–4591 10.1073/pnas.1000097107 20571116 PMC3063589

[B11] De Filippo C. Cavalieri D. Di Paola M. Ramazzotti M. Poullet J. B. Massart S. Collini S. Pieraccini G. Lionetti P. (2010). Impact of diet in shaping gut microbiota revealed by a comparative study in children from Europe and rural Africa. Proc. Natl. Acad. Sci. U.S.A. 107, 14691–14696 10.1073/pnas.1005963107 20679230 PMC2930426

[B12] Derrien M. Collado M. C. Ben-Amor K. Salminen S. De Vos W. M. (2008). The Mucin degrader *Akkermansia muciniphila* is an abundant resident of the human intestinal tract. Appl. Environ. Microbiol. 74, 1646–1648 10.1128/AEM.01226-07 18083887 PMC2258631

[B13] Dicksved J. Halfvarson J. Rosenquist M. Jarnerot G. Tysk C. Apajalahti J. Engstrand L. Jansson J. K. (2008). Molecular analysis of the gut microbiota of identical twins with Crohn's disease. ISME J. 2, 716–727 10.1038/ismej.2008.37 18401439

[B14] Dominguez-Bello M. G. Costello E. K. Contreras M. Magris M. Hidalgo G. Fierer N. Knight R. (2010). Delivery mode shapes the acquisition and structure of the initial microbiota across multiple body habitats in newborns. Proc. Natl. Acad. Sci. U.S.A. 107, 11971–11975 10.1073/pnas.1002601107 20566857 PMC2900693

[B15] Faith J. J. McNulty N. P. Rey F. E. Gordon J. I. (2011). Predicting a human gut microbiota's response to diet in gnotobiotic mice. Science 333, 101–104 10.1126/science.1206025 21596954 PMC3303606

[B16] Favier C. F. De Vos W. M. Akkermans A. D. (2003). Development of bacterial and bifidobacterial communities in feces of newborn babies. Anaerobe 9, 219–229 10.1016/j.anaerobe.2003.07.001 16887708

[B17] Favier C. F. Vaughan E. E. De Vos W. M. Akkermans A. D. (2002). Molecular monitoring of succession of bacterial communities in human neonates. Appl. Environ. Microbiol. 68, 219–226 10.1128/AEM.68.1.219-226.2002 11772630 PMC126580

[B18] Frank D. N. St Amand A. L. Feldman R. A. Boedeker E. C. Harpaz N. Pace N. R. (2007). Molecular-phylogenetic characterization of microbial community imbalances in human inflammatory bowel diseases. Proc. Natl. Acad. Sci. U.S.A. 104, 13780–13785 10.1073/pnas.0706625104 17699621 PMC1959459

[B19] Gill S. R. Pop M. Deboy R. T. Eckburg P. B. Turnbaugh P. J. Samuel B. S. Gordon J. I. Relman D. A. Fraser-Liggett C. M. Nelson K. E. (2006). Metagenomic analysis of the human distal gut microbiome. Science 312, 1355–1359 10.1126/science.1124234 16741115 PMC3027896

[B20] Gonzalez R. Klaassens E. S. Malinen E. De Vos W. M. Vaughan E. E. (2008). Differential transcriptional response of *Bifidobacterium longum* to human milk, formula milk, and galactooligosaccharide. Appl. Environ. Microbiol. 74, 4686–4694 10.1128/AEM.00122-08 18539808 PMC2519361

[B21] Gosalbes M. J. Durban A. Pignatelli M. Abellan J. J. Jimenez-Hernandez N. Perez-Cobas A. E. Latorre A. Moya A. (2011). Metatranscriptomic approach to analyze the functional human gut microbiota. PLoS ONE 6:e17447 10.1371/journal.pone.0017447 21408168 PMC3050895

[B23] Guarner F. Malagelada J. R. (2003). Gut flora in health and disease. Lancet 361, 512–519 10.1016/S0140-6736(03)12489-0 12583961

[B24] Hildebrandt M. A. Hoffmann C. Sherrill-Mix S. A. Keilbaugh S. A. Hamady M. Chen Y. Y. Knight R. Ahima R. S. Bushman F. Wu G. D. (2009). High-fat diet determines the composition of the murine gut microbiome independently of obesity. Gastroenterology 137, 1716–1724, e1711–e1712. 10.1053/j.gastro.2009.08.042 19706296 PMC2770164

[B25] Jalanka-Tuovinen J. Salonen A. Nikkila J. Immonen O. Kekkonen R. Lahti L. Palva A. De Vos W. M. (2011). Intestinal microbiota in healthy adults: temporal analysis reveals individual and common core and relation to intestinal symptoms. PLoS ONE 6:e23035 10.1371/journal.pone.0023035 21829582 PMC3145776

[B26] Jansson J. Willing B. Lucio M. Fekete A. Dicksved J. Halfvarson J. Tysk C. Schmitt-Kopplin P. (2009). Metabolomics reveals metabolic biomarkers of Crohn's disease. PLoS ONE 4:e6386 10.1371/journal.pone.0006386 19636438 PMC2713417

[B27] Kang S. Denman S. E. Morrison M. Yu Z. Dore J. Leclerc M. McSweeney C. S. (2010). Dysbiosis of fecal microbiota in Crohn's disease patients as revealed by a custom phylogenetic microarray. Inflamm. Bowel Dis. 16, 2034–2042 10.1002/ibd.21319 20848492

[B28] Klaassens E. S. Ben-Amor K. Vriesema A. Vaughan E. E. De Vos W. (2011). The fecal bifidobacterial transcriptome of adults: a microarray approach. Gut Microbes 2, 217–226 10.4161/gmic.2.4.16798 21983068

[B29] Klaassens E. S. Boesten R. J. Haarman M. Knol J. Schuren F. H. Vaughan E. E. De Vos W. M. (2009). Mixed-species genomic microarray analysis of fecal samples reveals differential transcriptional responses of bifidobacteria in breast- and formula-fed infants. Appl. Environ. Microbiol. 75, 2668–2676 10.1128/AEM.02492-08 19286790 PMC2681671

[B30] Klaassens E. S. De Vos W. M. Vaughan E. E. (2007). Metaproteomics approach to study the functionality of the microbiota in the human infant gastrointestinal tract. Appl. Environ. Microbiol. 73, 1388–1392 10.1128/AEM.01921-06 17158612 PMC1828649

[B31] Knol J. Scholtens P. Kafka C. Steenbakkers J. Gro S. Helm K. Klarczyk M. Schopfer H. Bockler H. M. Wells J. (2005). Colon microflora in infants fed formula with galacto- and fructo-oligosaccharides: more like breast-fed infants. J. Pediatr. Gastroenterol. Nutr. 40, 36–42 15625424 10.1097/00005176-200501000-00007

[B32] Koenig J. E. Spor A. Scalfone N. Fricker A. D. Stombaugh J. Knight R. Angenent L. T. Ley R. E. (2011). Succession of microbial consortia in the developing infant gut microbiome. Proc. Natl. Acad. Sci. U.S.A. 108(Suppl. 1), 4578–4585 10.1073/pnas.1000081107 20668239 PMC3063592

[B33] Kolmeder C. A. De Been M. Nikkila J. Ritamo I. Matto J. Valmu L. Salojarvi J. Palva A. Salonen A. De Vos W. M. (2012). Comparative metaproteomics and diversity analysis of human intestinal microbiota testifies for its temporal stability and expression of core functions. PLoS ONE 7:e29913 10.1371/journal.pone.0029913 22279554 PMC3261163

[B34] Kovatcheva-Datchary P. Egert M. Maathuis A. Rajilic-Stojanovic M. De Graaf A. A. Smidt H. De Vos W. M. Venema K. (2009). Linking phylogenetic identities of bacteria to starch fermentation in an *in vitro* model of the large intestine by RNA-based stable isotope probing. Environ. Microbiol. 11, 914–926 10.1111/j.1462-2920.2008.01815.x 19128319

[B35] Kurokawa K. Itoh T. Kuwahara T. Oshima K. Toh H. Toyoda A. Takami H. Morita H. Sharma V. K. Srivastava T. P. Taylor T. D. Noguchi H. Mori H. Ogura Y. Ehrlich D. S. Itoh K. Takagi T. Sakaki Y. Hayashi T. Hattori M. (2007). Comparative metagenomics revealed commonly enriched gene sets in human gut microbiomes. DNA Res. 14, 169–181 10.1093/dnares/dsm018 17916580 PMC2533590

[B36] Ley R. E. Backhed F. Turnbaugh P. Lozupone C. A. Knight R. D. Gordon J. I. (2005). Obesity alters gut microbial ecology. Proc. Natl. Acad. Sci. U.S.A. 102, 11070–11075 10.1073/pnas.0504978102 16033867 PMC1176910

[B37] Ley R. E. Turnbaugh P. J. Klein S. Gordon J. I. (2006). Microbial ecology: human gut microbes associated with obesity. Nature 444, 1022–1023 10.1038/4441022a 17183309

[B38] Macfarlane G. T. Macfarlane S. (2011). Fermentation in the human large intestine: its physiologic consequences and the potential contribution of prebiotics. J. Clin. Gastroenterol. 45(Suppl.) S120–S127 10.1097/MCG.0b013e31822fecfe 21992950

[B39] Makivuokko H. Tiihonen K. Tynkkynen S. Paulin L. Rautonen N. (2010). The effect of age and non-steroidal anti-inflammatory drugs on human intestinal microbiota composition. Br. J. Nutr. 103, 227–234 10.1017/S0007114509991553 19703328

[B40] Manichanh C. Rigottier-Gois L. Bonnaud E. Gloux K. Pelletier E. Frangeul L. Nalin R. Jarrin C. Chardon P. Marteau P. Roca J. Dore J. (2006). Reduced diversity of faecal microbiota in Crohn's disease revealed by a metagenomic approach. Gut 55, 205–211 10.1136/gut.2005.073817 16188921 PMC1856500

[B41] Marcobal A. Barboza M. Froehlich J. W. Block D. E. German J. B. Lebrilla C. B. Mills D. A. (2010). Consumption of human milk oligosaccharides by gut-related microbes. J. Agric. Food Chem. 58, 5334–5340 10.1021/jf9044205 20394371 PMC2866150

[B42] Marcobal A. Barboza M. Sonnenburg E. D. Pudlo N. Martens E. C. Desai P. Lebrilla C. B. Weimer B. C. Mills D. A. German J. B. Sonnenburg J. L. (2011). Bacteroides in the infant gut consume milk oligosaccharides via mucus-utilization pathways. Cell Host Microbe 10, 507–514 10.1016/j.chom.2011.10.007 22036470 PMC3227561

[B43] Mariat D. Firmesse O. Levenez F. Guimaraes V. Sokol H. Dore J. Corthier G. Furet J. P. (2009). The Firmicutes/Bacteroidetes ratio of the human microbiota changes with age. BMC Microbiol. 9, 123 10.1186/1471-2180-9-123 19508720 PMC2702274

[B44] McNulty N. P. Yatsunenko T. Hsiao A. Faith J. J. Muegge B. D. Goodman A. L. Henrissat B. Oozeer R. Cools-Portier S. Gobert G. Chervaux C. Knights D. Lozupone C. A. Knight R. Duncan A. E. Bain J. R. Muehlbauer M. J. Newgard C. B. Heath A. C. Gordon J. I. (2011). The impact of a consortium of fermented milk strains on the gut microbiome of gnotobiotic mice and monozygotic twins. Sci. Transl. Med. 3, 106ra106 10.1126/scitranslmed.3002701 22030749 PMC3303609

[B45] Monira S. Nakamura S. Gotoh K. Izutsu K. Watanabe H. Alam N. H. Endtz H. P. Cravioto A. Ali S. I. Nakaya T. Horii T. Iida T. Alam M. (2011). Gut microbiota of healthy and malnourished children in Bangladesh. Front. Microbiol. 2:228 10.3389/fmicb.2011.00228 22125551 PMC3221396

[B46] Monsen A. L. Refsum H. Markestad T. Ueland P. M. (2003). Cobalamin status and its biochemical markers methylmalonic acid and homocysteine in different age groups from 4 days to 19 years. Clin. Chem. 49, 2067–2075 10.1373/clinchem.2003.019869 14633879

[B47] Muegge B. D. Kuczynski J. Knights D. Clemente J. C. Gonzalez A. Fontana L. Henrissat B. Knight R. Gordon J. I. (2011). Diet drives convergence in gut microbiome functions across mammalian phylogeny and within humans. Science 332, 970–974 10.1126/science.1198719 21596990 PMC3303602

[B48] Murphy E. F. Cotter P. D. Hogan A. O'Sullivan O. Joyce A. Fouhy F. Clarke S. F. Marques T. M. O'Toole P. W. Stanton C. Quigley E. M. Daly C. Ross P. R. O'Doherty R. M. Shanahan F. (2012). Divergent metabolic outcomes arising from targeted manipulation of the gut microbiota in diet-induced obesity. Gut. [Epub ahead of print]. 10.1136/gutjnl-2011-300705 22345653

[B49] Nadal I. Santacruz A. Marcos A. Warnberg J. Garagorri M. Moreno L. A. Martin-Matillas M. Campoy C. Marti A. Moleres A. Delgado M. Veiga O. L. Garcia-Fuentes M. Redondo C. G. Sanz Y. (2009). Shifts in clostridia, bacteroides and immunoglobulin-coating fecal bacteria associated with weight loss in obese adolescents. Int. J. Obes. (Lond.) 33, 758–767 10.1038/ijo.2008.260 19050675

[B50] Nanthakumar N. N. Fusunyan R. D. Sanderson I. Walker W. A. (2000). Inflammation in the developing human intestine: a possible pathophysiologic contribution to necrotizing enterocolitis. Proc. Natl. Acad. Sci. U.S.A. 97, 6043–6048 10.1073/pnas.97.11.6043 10823949 PMC18555

[B51] Niers L. E. M. Martin R. Rijkers G. T. Sengers F. Timmerman H. M. van Uden N. O. Smidt H. Kimpen J. L. L. Hoekstra M. O. (2009) The effects of selected probiotic strains on the development of eczema (The PandA study). Allergy 64, 1349–1358 10.1111/j.1398-9995.2009.02021.x 19392993

[B52] O'Connell Motherway M. Zomer A. Leahy S. C. Reunanen J. Bottacini F. Claesson M. J. O'Brien F. Flynn K. Casey P. G. Munoz J. A. Kearney B. Houston A. M. O'Mahony C. Higgins D. G. Shanahan F. Palva A. De Vos W. M. Fitzgerald G. F. Ventura M. O'Toole P. W. Van Sinderen D. (2011). Functional genome analysis of *Bifidobacterium breve* UCC2003 reveals type IVb tight adherence (Tad) pili as an essential and conserved host-colonization factor. Proc. Natl. Acad. Sci. U.S.A. 108, 11217–11222 10.1073/pnas.1105380108 21690406 PMC3131351

[B53] O'Hara A. M. Shanahan F. (2006). The gut flora as a forgotten organ. EMBO Rep. 7, 688–693 10.1038/sj.embor.7400731 16819463 PMC1500832

[B54] Palmer C. Bik E. M. Digiulio D. B. Relman D. A. Brown P. O. (2007). Development of the human infant intestinal microbiota. PLoS Biol. 5:e177 10.1371/journal.pbio.0050177 17594176 PMC1896187

[B55] Penders J. Thijs C. Vink C. Stelma F. F. Snijders B. Kummeling I. Van Den Brandt P. A. Stobberingh E. E. (2006). Factors influencing the composition of the intestinal microbiota in early infancy. Pediatrics 118, 511–521 10.1542/peds.2005-2824 16882802

[B56] Pflughoeft K. J. Versalovic J. (2012). Human microbiome in health and disease. Annu. Rev. Pathol. 7, 99–122 10.1146/annurev-pathol-011811-132421 21910623

[B57] Png C. W. Linden S. K. Gilshenan K. S. Zoetendal E. G. McSweeney C. S. Sly L. I. McGuckin M. A. Florin T. H. (2010). Mucolytic bacteria with increased prevalence in IBD mucosa augment *in vitro* utilization of mucin by other bacteria. Am. J. Gastroenterol. 105, 2420–2428 10.1038/ajg.2010.281 20648002

[B58] Qin J. Li R. Raes J. Arumugam M. Burgdorf K. S. Manichanh C. Nielsen T. Pons N. Levenez F. Yamada T. Mende D. R. Li J. Xu J. Li S. Li D. Cao J. Wang B. Liang H. Zheng H. Xie Y. Tap J. Lepage P. Bertalan M. Batto J. M. Hansen T. Le Paslier D. Linneberg A. Nielsen H. B. Pelletier E. Renault P. Sicheritz-Ponten T. Turner K. Zhu H. Yu C. Jian M. Zhou Y. Li Y. Zhang X. Qin N. Yang H. Wang J. Brunak S. Dore J. Guarner F. Kristiansen K. Pedersen O. Parkhill J. Weissenbach J. Meta H. I. T. C. Bork P. Ehrlich S. D. (2010). A human gut microbial gene catalogue established by metagenomic sequencing. Nature 464, 59–65 10.1038/nature08821 20203603 PMC3779803

[B59] Rajilic-Stojanovic M. Heilig H. G. Molenaar D. Kajander K. Surakka A. Smidt H. De Vos W. M. (2009). Development and application of the human intestinal tract chip, a phylogenetic microarray: analysis of universally conserved phylotypes in the abundant microbiota of young and elderly adults. Environ. Microbiol. 11, 1736–1751 10.1111/j.1462-2920.2009.01900.x 19508560 PMC2784037

[B60] Rajilic-Stojanovic M. Shanahan F. Guarner F. de Vos W. M. (2012). Phylogenetic analysis of dysbiosis in ulcerative colitis in relapse and remission. Gut. (in press). 10.1097/MIB.0b013e31827fec6d 23385241

[B61] Roediger W. E. Moore J. Babidge W. (1997). Colonic sulfide in pathogenesis and treatment of ulcerative colitis. Dig. Dis. Sci. 42, 1571–1579 10.1023/A:1018851723920 9286219

[B62] Rooijers K. Kolmeder C. Juste C. Dore J. De Been M. Boeren S. Galan P. Beauvallet C. De Vos W. M. Schaap P. J. (2011). An iterative workflow for mining the human intestinal metaproteome. BMC Genomics 12, 6 10.1186/1471-2164-12-6 21208423 PMC3023752

[B63] Santacruz A. Collado M. C. Garcia-Valdes L. Segura M. T. Martin-Lagos J. A. Anjos T. Marti-Romero M. Lopez R. M. Florido J. Campoy C. Sanz Y. (2010). Gut microbiota composition is associated with body weight, weight gain and biochemical parameters in pregnant women. Br. J. Nutr. 104, 83–92 10.1017/S0007114510000176 20205964

[B64] Santacruz A. Marcos A. Warnberg J. Marti A. Martin-Matillas M. Campoy C. Moreno L. A. Veiga O. Redondo-Figuero C. Garagorri J. M. Azcona C. Delgado M. Garcia-Fuentes M. Collado M. C. Sanz Y. Group E. S. (2009). Interplay between weight loss and gut microbiota composition in overweight adolescents. Obesity (Silver Spring) 17, 1906–1915 10.1038/oby.2009.112 19390523

[B65] Scholtens P. A. Oozeer R. Martin R. Amor K. B. Knol J. (2012). The early settlers: intestinal microbiology in early life. Annu. Rev. Food Sci. Technol. 3, 425–447 10.1146/annurev-food-022811-101120 22224552

[B66] Schwartz S. Friedberg I. Ivanov I. V. Davidson L. A. Goldsby J. S. Dahl D. B. Herman D. Wang M. Donovan S. M. Chapkin R. (2012). A metagenomic study of diet-dependent interaction between gut microbiota and host in infants reveals differences in immune response. Genome Biol. 13, R32 10.1186/gb-2012-13-4-r32 22546241 PMC3446306

[B67] Schwiertz A. Taras D. Schafer K. Beijer S. Bos N. A. Donus C. Hardt P. D. (2010). Microbiota and SCFA in lean and overweight healthy subjects. Obesity (Silver Spring) 18, 190–195 10.1038/oby.2009.167 19498350

[B68] Sekirov I. Russell S. L. Antunes L. C. Finlay B. B. (2010). Gut microbiota in health and disease. Physiol. Rev. 90, 859–904 10.1152/physrev.00045.2009 20664075

[B69] Sela D. A. Chapman J. Adeuya A. Kim J. H. Chen F. Whitehead T. R. Lapidus A. Rokhsar D. S. Lebrilla C. B. German J. B. Price N. P. Richardson P. M. Mills D. A. (2008). The genome sequence of *Bifidobacterium longum* subsp. infantis reveals adaptations for milk utilization within the infant microbiome. Proc. Natl. Acad. Sci. U.S.A. 105, 18964–18969 10.1073/pnas.0809584105 19033196 PMC2596198

[B70] Sjogren Y. M. Jenmalm M. C. Bottcher M. F. Bjorksten B. Sverremark-Ekstrom E. (2009). Altered early infant gut microbiota in children developing allergy up to 5 years of age. Clin. Exp. Allergy 39, 518–526 10.1111/j.1365-2222.2008.03156.x 19220322

[B71] Tasse L. Bercovici J. Pizzut-Serin S. Robe P. Tap J. Klopp C. Cantarel B. L. Coutinho P. M. Henrissat B. Leclerc M. Dore J. Monsan P. Remaud-Simeon M. Potocki-Veronese G. (2010). Functional metagenomics to mine the human gut microbiome for dietary fiber catabolic enzymes. Genome Res. 20, 1605–1612 10.1101/gr.108332.110 20841432 PMC2963823

[B72] Tedelind S. Westberg F. Kjerrulf M. Vidal A. (2007). Anti-inflammatory properties of the short-chain fatty acids acetate and propionate: a study with relevance to inflammatory bowel disease. World J. Gastroenterol. 13, 2826–2832 17569118 10.3748/wjg.v13.i20.2826 PMC4395634

[B73] Turnbaugh P. J. Hamady M. Yatsunenko T. Cantarel B. L. Duncan A. Ley R. E. Sogin M. L. Jones W. J. Roe B. A. Affourtit J. P. Egholm M. Henrissat B. Heath A. C. Knight R. Gordon J. I. (2009a). A core gut microbiome in obese and lean twins. Nature 457, 480–484 10.1038/nature07540 19043404 PMC2677729

[B75] Turnbaugh P. J. Ley R. E. Mahowald M. A. Magrini V. Mardis E. R. Gordon J. I. (2006). An obesity-associated gut microbiome with increased capacity for energy harvest. Nature 444, 1027–1031 10.1038/nature05414 17183312

[B76] Turnbaugh P. J. Quince C. Faith J. J. McHardy A. C. Yatsunenko T. Niazi F. Affourtit J. Egholm M. Henrissat B. Knight R. Gordon J. I. (2010). Organismal, genetic, and transcriptional variation in the deeply sequenced gut microbiomes of identical twins. Proc. Natl. Acad. Sci. U.S.A. 107, 7503–7508 10.1073/pnas.1002355107 20363958 PMC2867707

[B74] Turnbaugh P. J. Ridaura V. K. Faith J. J. Rey F. E. Knight R. Gordon J. I. (2009b). The effect of diet on the human gut microbiome: a metagenomic analysis in humanized gnotobiotic mice. Sci. Transl. Med. 1, 6ra14 10.1126/scitranslmed.3000322 20368178 PMC2894525

[B77] Turroni F. Peano C. Pass D. A. Foroni E. Severgnini M. Claesson M. J. Kerr C. Hourihane J. Murray D. Fuligni F. Gueimonde M. Margolles A. De Bellis G. O'Toole P. W. Van Sinderen D. Marchesi J. R. Ventura M. (2012). Diversity of Bifidobacteria within the infant gut microbiota. PLoS ONE 7:e36957 10.1371/journal.pone.0036957 22606315 PMC3350489

[B78] Vaishampayan P. A. Kuehl J. V. Froula J. L. Morgan J. L. Ochman H. Francino M. P. (2010). Comparative metagenomics and population dynamics of the gut microbiota in mother and infant. Genome Biol. Evol. 2, 53–66 10.1093/gbe/evp057 20333224 PMC2839348

[B79] Verberkmoes N. C. Russell A. L. Shah M. Godzik A. Rosenquist M. Halfvarson J. Lefsrud M. G. Apajalahti J. Tysk C. Hettich R. L. Jansson J. K. (2009). Shotgun metaproteomics of the human distal gut microbiota. ISME J. 3, 179–189 10.1038/ismej.2008.108 18971961

[B80] Vijay-Kumar M. Aitken J. D. Carvalho F. A. Cullender T. C. Mwangi S. Srinivasan S. Sitaraman S. V. Knight R. Ley R. E. Gewirtz A. T. (2010). Metabolic syndrome and altered gut microbiota in mice lacking Toll-like receptor 5. Science 328, 228–231 10.1126/science.1179721 20203013 PMC4714868

[B81] Willing B. P. Dicksved J. Halfvarson J. Andersson A. F. Lucio M. Zheng Z. Jarnerot G. Tysk C. Jansson J. K. Engstrand L. (2010). A pyrosequencing study in twins shows that gastrointestinal microbial profiles vary with inflammatory bowel disease phenotypes. Gastroenterology 139, 1844–1854, e1841. 10.1053/j.gastro.2010.08.049 20816835

[B82] Wu G. D. Chen J. Hoffmann C. Bittinger K. Chen Y. Y. Keilbaugh S. A. Bewtra M. Knights D. Walters W. A. Knight R. Sinha R. Gilroy E. Gupta K. Baldassano R. Nessel L. Li H. Bushman F. D. Lewis J. D. (2011). Linking long-term dietary patterns with gut microbial enterotypes. Science 334, 105–108 10.1126/science.1208344 21885731 PMC3368382

[B83] Yatsunenko T. Rey F. E. Manary M. J. Trehan I. Dominguez-Bello M. G. Contreras M. Magris M. Hidalgo G. Baldassano R. N. Anokhin A. P. Heath A. C. Warner B. Reeder J. Kuczynski J. Caporaso J. G. Lozupone C. A. Lauber C. Clemente J. C. Knights D. Knight R. Gordon J. I. (2012). Human gut microbiome viewed across age and geography. Nature 486, 222–227 10.1038/nature11053 22699611 PMC3376388

[B84] Zhang H. Dibaise J. K. Zuccolo A. Kudrna D. Braidotti M. Yu Y. Parameswaran P. Crowell M. D. Wing R. Rittmann B. E. Krajmalnik-Brown R. (2009). Human gut microbiota in obesity and after gastric bypass. Proc. Natl. Acad. Sci. U.S.A. 106, 2365–2370 10.1073/pnas.0812600106 19164560 PMC2629490

[B85] Zoetendal E. G. Raes J. Van Den Bogert B. Arumugam M. Booijink C. C. Troost F. J. Bork P. Wels M. De Vos W. M. Kleerebezem M. (2012). The human small intestinal microbiota is driven by rapid uptake and conversion of simple carbohydrates. ISME J. 6, 1415–1426 10.1038/ismej.2011.212 22258098 PMC3379644

[B86] Zoetendal E. G. Rajilic-Stojanovic M. De Vos W. M. (2008). High-throughput diversity and functionality analysis of the gastrointestinal tract microbiota. Gut 57, 1605–1615 10.1136/gut.2007.133603 18941009

